# Screening and evaluation of therapeutic candidates with vascular protective effects in zebrafish models of diabetic retinopathy

**DOI:** 10.1038/s41598-025-20272-7

**Published:** 2025-10-15

**Authors:** Yujin Lee, Young min Cha, Jaewook Yang

**Affiliations:** 1 Department of Ophthalmology, Inje University Busan Paik Hospital, 75 Bokji-ro, Busanjin-gu, Busan, South Korea; 2 EYEBIOKOREA, 81, Jinsa-ro 83beon-gil, Busanjin-gu, Busan, South Korea; 3 Department of Dentistry, Inje University Busan Paik Hospital, 75 Bokji-ro, Busanjin-gu, Busan, South Korea

**Keywords:** Diabetic retinopathy (DR), Non-Proliferative diabetic retinopathy (NPDR), Zebrafish, Embryonic toxicity, Drug screening, Vascular protection, Tie2, Angiopoietin-1, Vascular endothelial growth factor (VEGF), Diseases, Drug discovery, Endocrinology, Medical research, Molecular biology

## Abstract

We evaluated therapeutic peptide candidates for diabetic retinopathy (DR) using a zebrafish model. Three peptides, designed from a type II collagen-derived sequence, were evaluated for toxicity and vascular protective effects. Peptide 1 demonstrated favorable physicochemical stability, low toxicity (> 90% survival), and vascular protective activity. In contrast, Peptides 2 and 3 showed increased toxicity and morphological abnormalities at higher concentrations, limiting their potential utility. In a hyperglycemia-induced zebrafish DR model, Peptide 1 (100–200 µg/ml) reduced retinal vessel thickness with efficacy comparable to aflibercept. Molecular analysis by RT-PCR indicated that Peptide 1 suppressed vascular endothelial growth factor (VEGF) expression and enhanced Tie2 and Angiopoietin-1 (Ang-1) expression, suggesting a role in vascular stabilization. These findings establish zebrafish as a cost-effective and rapid screening platform for early-stage DR drug discovery. These findings support zebrafish as a cost-effective platform for early-stage diabetic retinopathy drug discovery and highlight Peptide 1 as a promising candidate for non-proliferative DR, providing a rationale for further optimization and mechanistic studies toward clinical translation.

## Introduction

Diabetic retinopathy (DR) is a major microvascular complication of diabetes and a leading cause of vision loss in working-age adults. Based on its progression, DR is classified into non-proliferative diabetic retinopathy (NPDR) and proliferative diabetic retinopathy (PDR)^1–3^. NPDR progresses through distinct stages: Stage 1 involves microaneurysms and increased vascular permeability, Stage 2 includes microvascular occlusion and localized ischemia, and Stage 3, or severe NPDR, is marked by extensive occlusion and upregulation of vascular endothelial growth factor(VEGF)^4,5^. If left untreated, NPDR can progress to PDR, where abnormal neovascularization leads to retinal and vitreous hemorrhages or retinal detachment, resulting in irreversible vision loss^6^. Early-stage DR is often asymptomatic^7^and although strict glycemic control slows progression there is no fundamental cure. Anti-VEGF intravitreal injections are the current standard treatment for PDR^8,9^but they involve infection risks, treatment burden, and frequent administration. Moreover, no specific therapies are approved for NPDR, which accounts for approximately 80% of DR cases^10–12^. These limitations highlight the urgent need for novel pharmacologic strategies targeting early stages of the disease.

The retina is one of the most metabolically active tissues in the human body, relying on the retinal vasculature to supply blood and oxygen to its inner two-thirds, thereby supporting normal visual function^13,14^. Tight junctions between retinal endothelial cells (ECs) form the inner blood-retinal barrier (BRB), which regulates the selective exchange of materials and prevents harmful substances from entering the neural retina^15–17^. This structural barrier plays a critical role in meeting the retina’s metabolic demands and maintaining neural homeostasis^18^. Hyperglycemia disrupts this barrier through inflammation and oxidative stress, causing vascular leakage, hypoxia, and retinal capillary loss^19–22^. The detachment of surrounding support cells further destabilizes blood vessels, resulting in retinal capillary non-perfusion and promoting the progression to PDRa major cause of vision loss, often accompanied by diabetic macular edema (DME)^23–25^. Hyperglycemia also increases vascular permeability through multiple mechanisms, contributing to retinal edema and inflammation^26^. Among the key regulators of vascular integrity is the Angiopoietin/Tie2 (Ang/Tie2) signaling pathway. In particular, Angiopoietin-1 (Ang-1), Tie2, and VEGF play critical roles in maintaining vascular stability and modulating angiogenesis in diabetic retinopathy^27,28^. Ang-1 binds to Tie2 to promote EC survival and BRB integrity, while activating downstream pathways such as AKT and MAPK, enhancing eNOS expression and nitric oxide (NO) production, and reducing ICAM-1 expression^29,30^. However, under diabetic conditions, inflammatory cytokines weaken tight junctions and increase BRB permeability^27^. In diabetic retinopathy, expression of Angiopoietin-2 (Ang-2) is upregulated, which antagonizes Tie2 activation and leads to vascular destabilization^31–33^. he resulting reduction in Tie2 signaling promotes vascular leakage, inflammation, and hypoxia, accelerating disease progression^34,35^. Concurrently, VEGF expression is elevated in response to retinal hypoxia and hyperglycemia, driving abnormal neovascularization and further compromising BRB integrity^36,37^. Therefore, strategies aimed at protecting the BRB, reducing vascular leakage, and inhibiting inflammation represent promising therapeutic approaches for slowing the progression of diabetic retinopathy and preventing vision-threatening complications^27,38^.

Peptides are gaining attention as therapeutic agents due to their high specificity, low toxicity, and favorable safety in vivo^39–41^. Clinically, peptide-based drugs such as insulin and glucagon-like peptide-1 (GLP-1) have shown efficacy in treating diabetes and DR^42,43^. In previous studies, a peptide called Hyp-GQDGLAGPK, derived from the N-terminal propeptide of type II collagen and synthesized using cartilage-derived extracellular matrix (CDECM), demonstrated anti-angiogenic and anti-inflammatory effects, including inhibition of corneal neovascularization (CNV) and stabilization of the tear film in a dry eye mouse model^44–47^. Building on these findings, a series of structurally modified peptide derivatives was designed to improve solubility and stability. From these candidates, three peptides (Peptides 1–3) were selected based on prior screening results and a previously filed patent (Application No. 10-2020-0060397). These peptides were designed with potential anti-inflammatory properties, suggesting applicability in inflammatory ocular diseases. Since inflammation and vascular instability are central to the pathogenesis of DR, they were expected to exert vascular-protective effects by modulating inflammatory pathways. Therefore, evaluating their efficacy in a zebrafish DR model may provide insights into their potential as early-stage therapeutic agents^48,49^.

Zebrafish are widely used as experimental models for ophthalmic disease research and drug screening due to their morphological and physiological similarities to the human retina, as well as their conserved mechanisms for glucose homeostasis^49,50^. Zebrafish embryos and adults possess key metabolic organs, including the liver, kidney, and blood-brain barrier, allowing for in vivo assessment of drug absorption, targeting, toxicity, and mechanism of action^51,52^. Their transparent embryos and rapid development enable high-throughput imaging of vascular structures with strong statistical reliability, making them an economical and efficient alternative to rodent models^53–55^. Previous studies have established a zebrafish model of DR induced by hyperglycemia, which can detect early-stage NPDR^49^. Compared to traditional rodent models, which often require disease progression before efficacy can be assessed, zebrafish models allow for earlier detection of therapeutic effects, particularly at the NPDR stage, thereby saving both time and cost in drug development^49,53^. This has led to their increased use as an alternative animal model for evaluating DR pathogenesis and treatment strategies^49^.

In this study, we aim to use zebrafish to evaluate the embryotoxicity of peptide, determine their effective doses and assess their vascular protection and stabilization effects in the diabetic retinopathy zebrafish model. Through this approach, we seek to identify compounds that demonstrate therapeutic effects.

## Result

### Toxicological evaluation of peptide 1 in zebrafish embryos

**Fig. 1:**
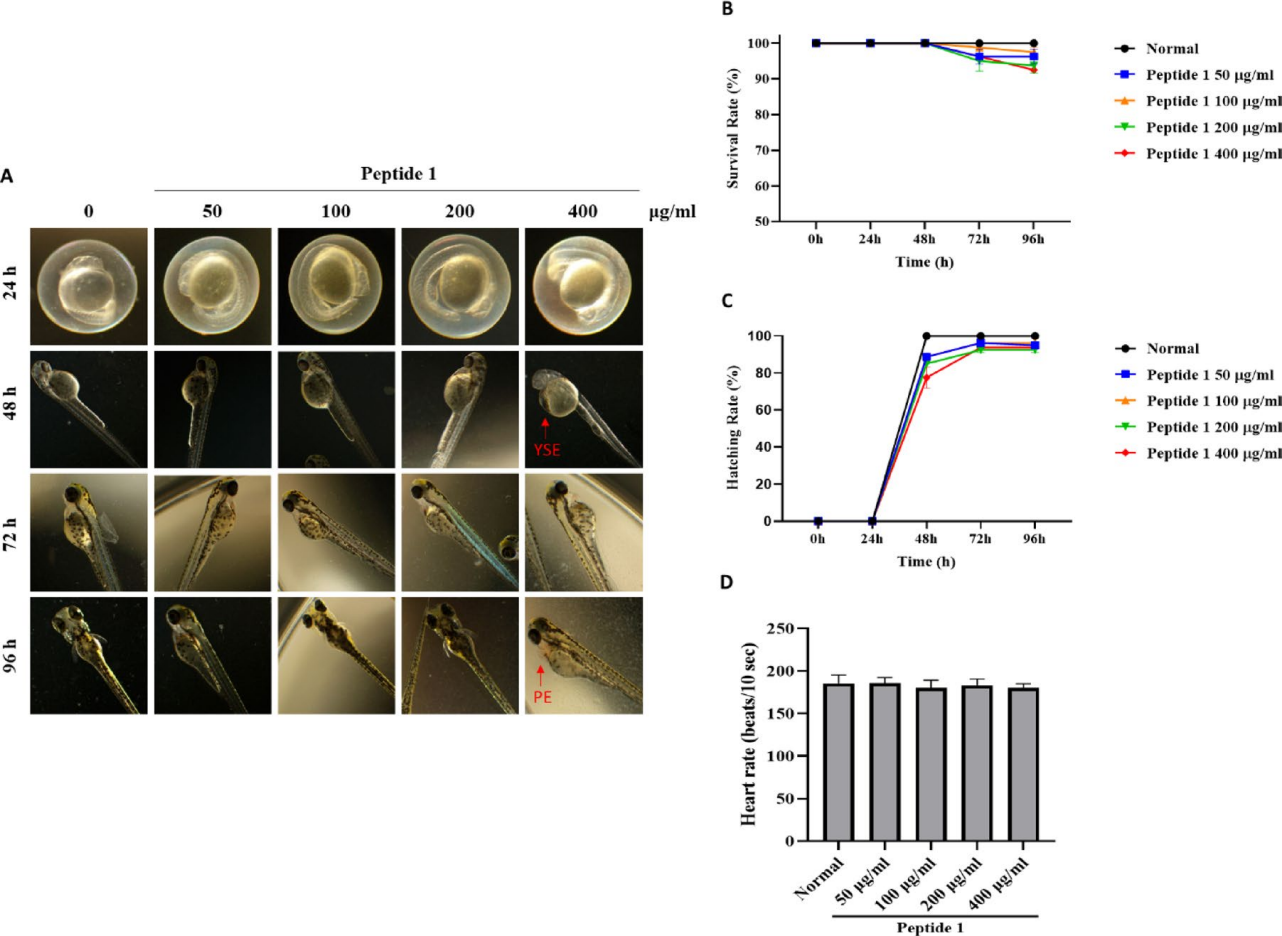
Effect of peptide 1 on the embryonic development of zebrafish. (**A**) Representative images of zebrafish embryos exposed to Peptide 1 at concentrations of 50, 100, 200, and 400 µg/mL for 24, 48, 72, and 96 h. (**B**) Hatching rate and (**C**) survival rate were quantified at each time point (*n* = 40 embryos per group). (**D**) Heart rates were measured by counting beats over 1 min, repeated 10 times per group (*n* = 10 per group). Data are presented as mean ± standard deviation (SD) from two independent experiments.

Zebrafish embryos were exposed to peptide 1 at concentrations of 50, 100, 200, and 400 µg/ml, and developmental parameters were monitored up to 96 h post-fertilization (hpf). The survival rates at 96 hpf were 96.25%, 97.5%, 93.75%, and 92.5%, respectively, with no statistically significant reduction compared to the control group (Fig. 1A, B). Differentiation, which began at 48 hpf, proceeded normally, with corresponding rates of 95%, 96.25%, 92.5%, and 93.75% at 96 hpf (Fig. 1A, C). While most embryos developed without defects, minor morphological abnormalities were noted at higher concentrations. Specifically, the collapse of the fertilized egg (CPFE) was observed at 200 µg/ml, and yolk sac edema (YSE) and pericardial edema (PE) were observed at 400 µg/ml (Table 1). Embryonic heart rates remained within the normal range across all treatment groups (Fig. 1D), suggesting no detectable cardiotoxicity. These findings suggest that peptide 1 exhibits low developmental toxicity in zebrafish embryos at concentrations up to 400 µg/ml, with only mild abnormalities observed at higher doses.

**Table 1:** Morphological abnormally symptoms of peptide 1 (%).

%	CPFE	YSE	PE	BT	HE
Normal	0.0	0.0	0.0	0.0	0.0
50 µg/ml	0.0	0.0	0.0	0.0	0.0
100 µg/ml	0.0	0.0	0.0	0.0	0.0
200 µg/ml	1.3	0.0	0.0	0.0	0.0
400 µg/ml	0.0	1.3	3.8	0.0	0.0

The morphological abnormalities compared to the normal group are expressed as a percentage (%).

### Toxicological evaluation of peptide 2 in zebrafish embryos

**Fig. 2:**
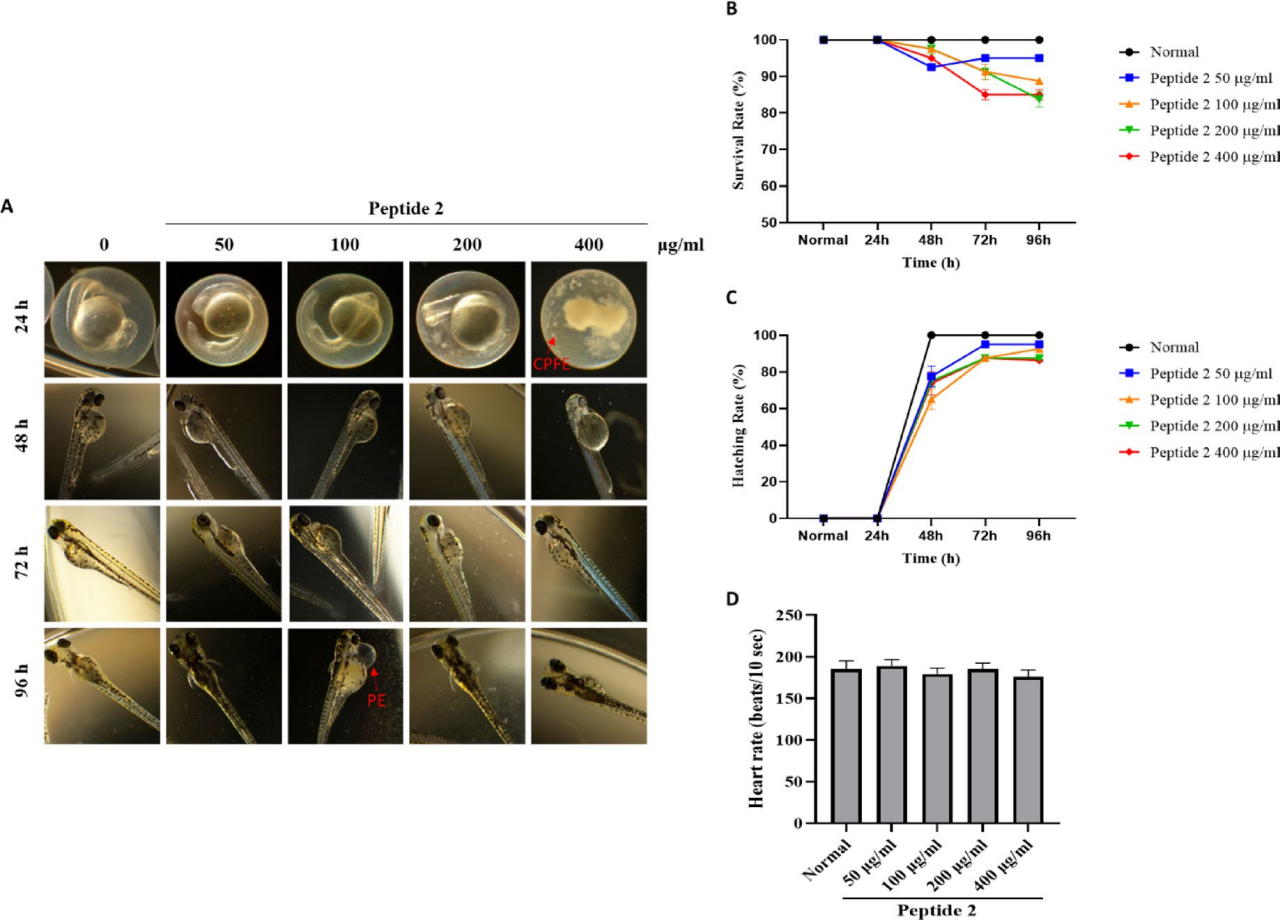
Effect of peptide 2 on the embryonic development of zebrafish. (**A**) Representative images of zebrafish embryos exposed to Peptide 2 at concentrations of 50, 100, 200, and 400 µg/mL for 24, 48, 72, and 96 h. (**B**) Hatching rates and (**C**) survival rates were quantified at each time point. (**D**) Morphological abnormalities were assessed in each treatment group (*n* = 40 per group). (**E**) Heart rates were measured by counting beats over 1 min, repeated 10 times per group (*n* = 10 per group). Data are presented as mean ± standard deviation (SD) from two independent experiments.

Zebrafish embryos were exposed to peptide 2 at concentrations of 50, 100, 200, and 400 µg/ml, and their development was monitored up to 96 h hpf. The survival rates at 96 h were 95%, 88.75%, 83.75%, and 85%, respectively, showing concentration-dependent decrease compared with the control group (Fig. 2A, B). Differentiation rates at 96 hpf were 95%, 92.5%, 87.5%, and 86.25%, respectively (Fig. 2A, C). At 48 hpf, when hatching typically occurs, delayed hatching was observed in all peptide 2-treated groups relative to the control. As shown in Table 2, morphological abnormalities, including collapse of the fertilized egg (CPFE) and pericardial edema (PE), were more frequent at higher concentrations. However, Heart rate measurements showed no significant differences between peptide 2-treated and control groups (Fig. 2D), indicating no detectable cardiotoxicity within the tested range. These data indicate that peptide 2 exhibited greater developmental toxicity than peptide 1, particularly at higher concentrations. Although the observed abnormalities were moderate, they warrant interpretation when considering the safety of to potential therapeutic applications.

**Table 2:** Morphological abnormally symptoms of peptide 2 (%).

%	CPFE	YSE	PE	BT	HE
Normal	0.0	0.0	0.0	0.0	0.0
50 µg/ml	0.0	0.0	2.5	0.0	0.0
100 µg/ml	2.5	0.0	0.0	0.0	0.0
200 µg/ml	0.0	0.0	2.5	0.0	0.0
400 µg/ml	2.5	0.0	5.0	0.0	0.0

The morphological abnormalities compared to the normal group are expressed as a percentage (%).

### Toxicological evaluation of peptide 3 in zebrafish embryo

**Fig. 3:**
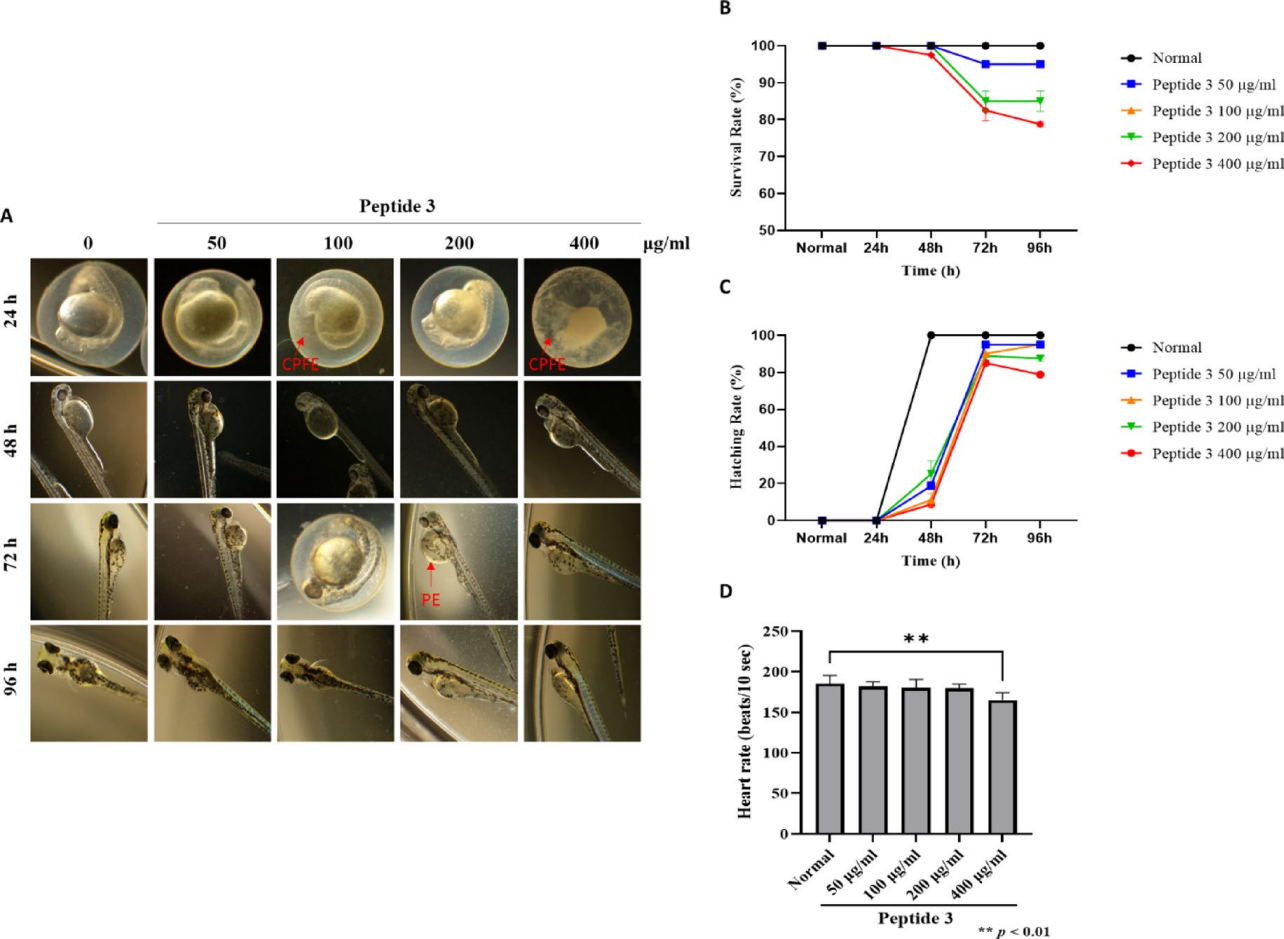
Effect of peptide 3 on the embryonic development of zebrafish. (**A**) Representative images of zebrafish embryos exposed to Peptide 3 at concentrations of 50, 100, 200, and 400 µg/mL for 24, 48, 72, and 96 h. (**B**) Hatching rates and (**C**) survival rates were quantified at each time point. (**D**) Morphological abnormalities were assessed in each treatment group (*n* = 40 per group). (**E**) Heart rates were measured by counting beats over 1 min, repeated 10 times per group (*n* = 10 per group). Data are presented as mean ± standard deviation (SD) from two independent experiments.

Zebrafish embryos were treated with peptide 3 at concentrations of 50, 100, 200, and 400 µg/ml. As shown in Fig. 3A and B, the survival rates at 96 h hpf were 95%, 95%, 85%, and 78.75%, respectively, while the differentiation rates were 95%, 95%, 87.5%, and 78.75% (Fig. 3C). The most pronounced reductions in both parameters were observed at 400 µg/ml, indicating a concentration-dependent effect. Across all concentrations, peptide 3 consistently yielded lower differentiation rates than the control group. Morphological abnormalities, including collapse of fertilized eggs (CPFE) at 100 and 400 µg/ml and yolk sac edema (YSE) at 200 µg/ml, were also noted (Table 3). Heart rate analysis revealed a significant decrease in the 400 µg/ml group (165.0 ± 9.1 beats/10 sec) compared to the control (185.4 ± 10 beats/10 sec), suggesting potential cardiotoxicity at this concentration (Fig. 3D). Taken together, these findings suggest that peptide 3 induces dose-dependent developmental toxicity in zebrafish embryos, particularly at higher concentrations, highlighting the need for further investigation of its safety.

**Table 3:** Morphological abnormally symptoms of peptide 3 (%).

%	CPFE	YSE	PE	BT	HE
Normal	0.0	0.0	0.0	0.0	0.0
50 µg/ml	0.0	0.0	0.0	0.0	0.0
100 µg/ml	2.5	0.0	0.0	0.0	0.0
200 µg/ml	0.0	2.5	0.0	0.0	0.0
400 µg/ml	5.0	0.0	0.0	0.0	0.0

The morphological abnormalities compared to the normal group are expressed as a percentage (%).

### Efficacy of peptides 1, 2, and 3 in the zebrafish DR model

**Fig. 4:**
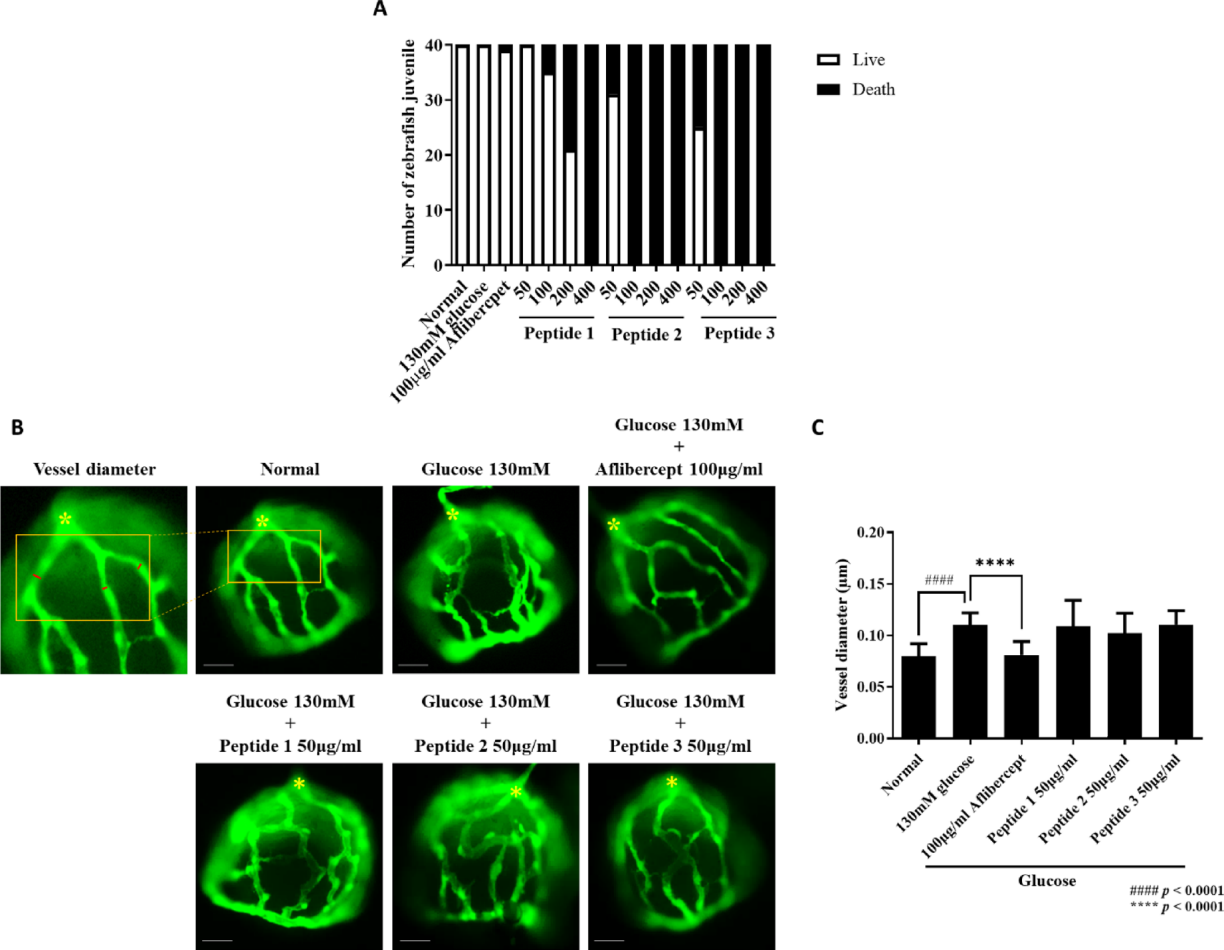
Effect of peptides 1, 2, and 3 on retinal vessel diameter in a zebrafish DR model. (**A**) Zebrafish larvae were treated with peptides 1, 2, and 3 at concentrations of 50, 100, 200, and 400 µg/ml from 3 to 6 dpf. The number of surviving larvae was recorded at 6 dpf. (**B**) Representative images of retinal vasculature were acquired using fluorescence microscopy (Leica DM2500). The yellow asterisk (*) indicates the optic nerve head, which served as the reference point for vessel diameter measurements. Vessel diameters were quantified by measuring 3 major retinal vessels radiating from the optic nerve using Image J software. Scale bar = 100 μm. (**C**) Quantification of retinal vessel diameters. Data are expressed as mean ± SD (*n* = 40 per group).####: *p* < 0.0001 vs. normal group; ****: *p* < 0.0001 vs. 130 mM glucose group.

In the zebrafish DR model established by high-glucose exposure (130 mM) from 3 to 6 dpf, peptides 1, 2, and 3 were administered at concentrations of 50–400 µg/ml. As shown in Fig. 4A, all zebrafish treated with 400 µg/ml of the peptides died, and Peptides 2 and 3 also caused marked mortality at 100 and 200 µg/ml. In contrast, Peptide 1 maintained survival up to 200 µg/ml. Based on these findings, efficacy evaluation was performed at 50 µg/ml, the highest non-lethal dose common to all groups (Fig. 4B). At this concentration, retinal vessel thickness was significantly increased in glucose-treated zebrafish compared with the normal control, and aflibercept (100 µg/ml) effectively reduced vessel thickness, consistent with previous findings (Lee & Yang, 2021). However, Peptides 1, 2, and 3 did not significantly alter retinal vascular morphology relative to the glucose-only group (Fig. 4C). Overall, these data indicate that Peptides 2 and 3 were associated with higher toxicity, whereas Peptide 1 showed comparatively favorable safety. Further studies evaluating Peptide 1 at safe but higher concentrations will be required to clarify its potential therapeutic efficacy relative to aflibercept.

### Efficacy of peptide 1 at different concentrations without observed toxicity

**Fig. 5:**
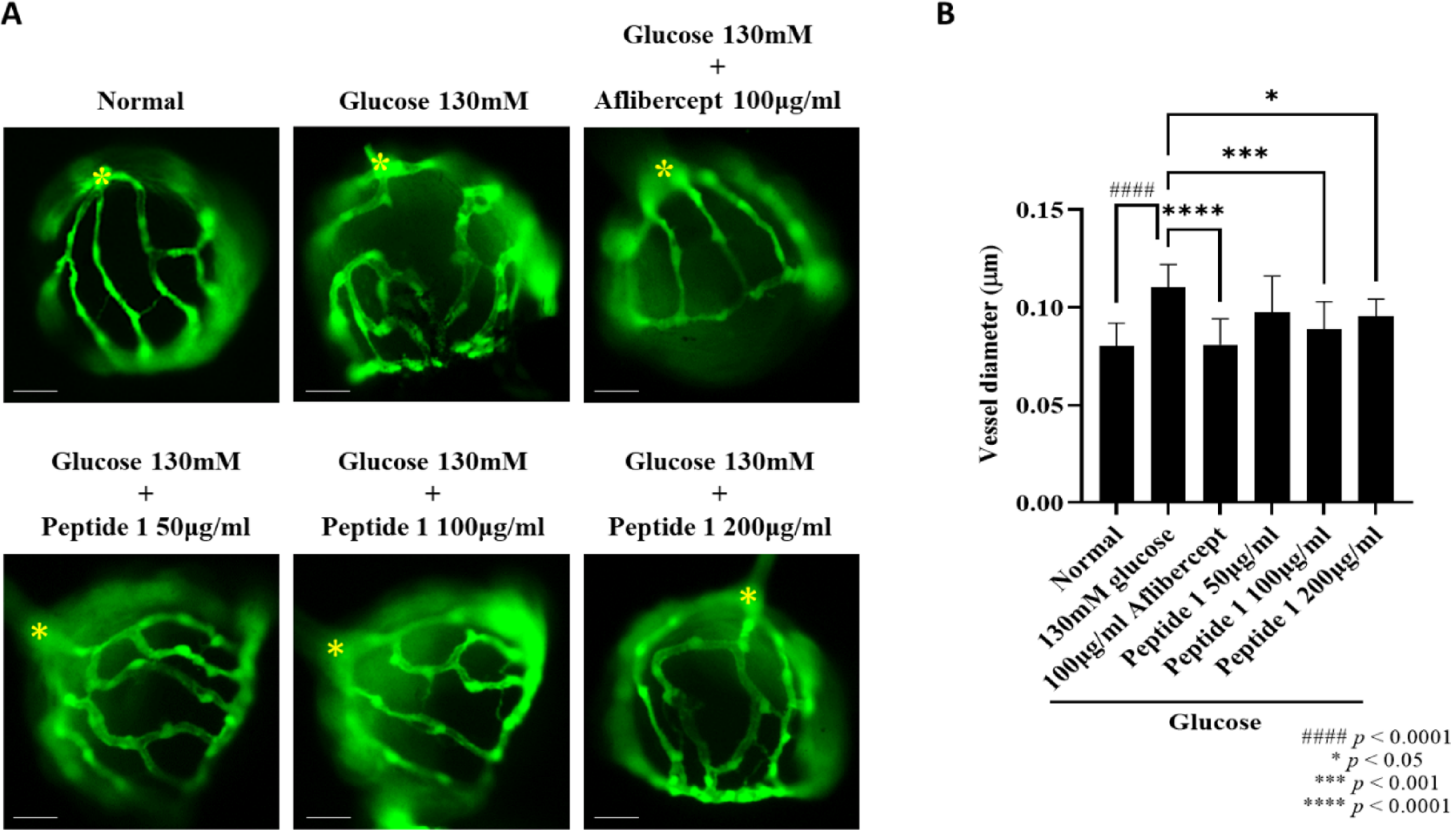
The effect of peptide 1 on retinal vessels in a zebrafish diabetic retinopathy model. (**A**) Zebrafish larvae were treated with peptide 1 at concentrations of 50, 100, and 200 µg/ml from 3 to 6 dpf. Representative fluorescent images of zebrafish hyaloid-retinal vessels are shown. Scale bar = 100 μm. (**B**) Retinal vessel diameters were quantified at three major vessels radiating from the optic nerve using Image J software. Data are expressed as mean ± SD (*n* = 40 per group). ####: *p* < 0.0001 vs. normal group. * and ****: *p* < 0.05 and *p* < 0.0001 vs. 130 mM glucose group.

Since Peptides 2 and 3 caused complete mortality at concentrations above 100 µg/ml, subsequent efficacy testing was focused on Peptide 1. In the zebrafish DR model, Peptide 1 was administered at 50, 100, and 200 µg/ml. Treatment with 100 and 200 µg/ml significantly reduced retinal vessel thickness to levels comparable to aflibercept (Fig. 5A, B). However, partial mortality was observed at 200 µg/ml (Fig. 4A), indicating a narrow safety margin at this dose. Based on these findings, 100 µg/ml was identified as the optimal concentration, showing significant efficacy while avoiding detectable toxicity.

### Efficacy of peptide 1 in vascular stability

**Fig. 6:**
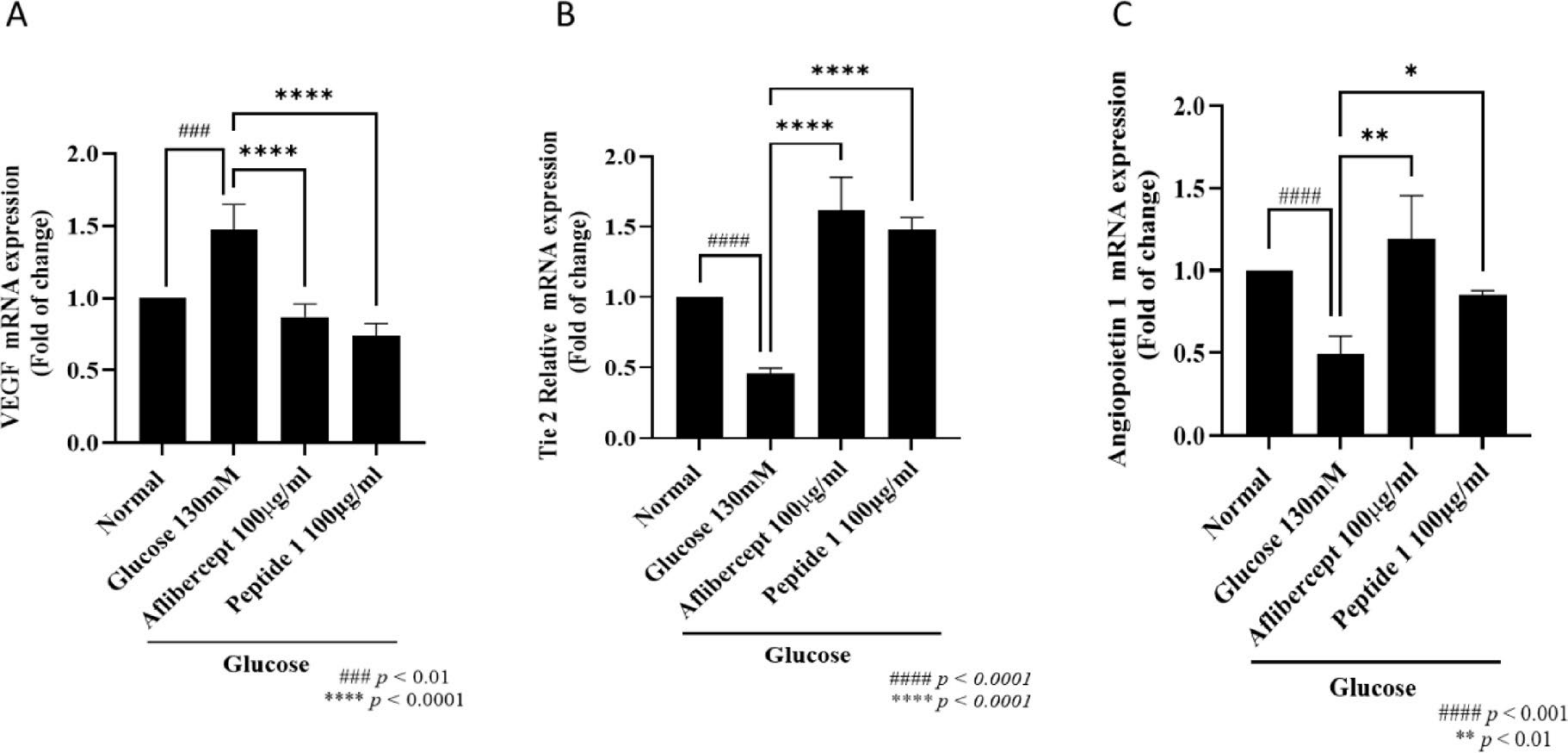
The effect of peptide 1 on mRNA expression levels measured by RT-PCR in zebrafish. Zebrafish larvae were treated with peptide 1 at concentrations of 50 and 200 µg/ml from 3 dpf to 6 dpf under high glucose conditions. mRNA levels of VEGF (**A**), Tie2 (**B**), and Angiopoietin-1 (**C**) were measured by RT-PCR. ####: *p* < 0.0001 vs. normal group. *, **, and ****: *p* < 0.05, *p* < 0.01, and *p* < 0.0001 vs. 130 mM glucose group.

To assess vascular stability, which is closely associated with the pathogenesis of DR, the expression of VEGF, Tie2, and Angiopoietin-1 was analyzed. As shown in Fig. 6A, VEGF mRNA levels were elevated under high-glucose conditions compared with the normal group, and this increase was significantly reduced by aflibercept (100 µg/ml). Treatment with Peptide 1 (100 µg/ml) resulted in a similar reduction in VEGF expression. In parallel, the downregulation of Tie2 and Angiopoietin-1 observed under high-glucose conditions was partially restored by both aflibercept and Peptide 1 (Fig. 6B, C).

## Discussion

This study evaluated the developmental toxicity and therapeutic potential of three candidate peptides (Peptide 1, Peptide 2, and Peptide 3) for DR treatment using a zebrafish model. The embryonic toxicity assessment indicated that Peptide 1 maintained high survival rates (> 90%) and normal heartbeats, suggesting relatively low toxicity. In contrast, Peptides 2 and 3 exhibited concentration-dependent toxicity, with reduced survival and morphological abnormalities, most notably at 400 µg/ml of Peptide 3. Evaluation of retinal vascular changes further reflected these differences. All three peptides were lethal at 400 µg/ml, and Peptides 2 and 3 caused marked mortality at 100–200 µg/ml, which precluded efficacy testing at these concentrations. By contrast, Peptide 1 preserved survival up to 200 µg/ml and significantly reduced hyperglycemia-induced retinal vessel thickening in the zebrafish DR model. This reduction was comparable to that observed with aflibercept, suggesting that Peptide 1 may achieve a more favorable balance between safety and efficacy. The distinct toxicity and efficacy among the peptides likely reflect differences in their physicochemical properties, such as solubility, aggregation propensity, and susceptibility to proteolytic degradation. Peptide 1 exhibited greater solubility and stability with a lower tendency to aggregate, which may have contributed to its reduced toxicity and sustained vascular protective effects. By contrast, Peptide 3 showed reduced solubility and a higher aggregation propensity, which could increase membrane interactions and toxicity^56,57^. Meanwhile, the shorter length of Peptide 2 may render it more susceptible to proteolytic degradation, potentially generating non-specific fragments that reduce efficacy and contribute to toxicity. Peptide instability and rapid degradation have been recognized as major challenges in the development of therapeutic peptides^58,59^. Although detailed structural analyses were not conducted in this study, these physicochemical considerations are consistent with well-established determinants of peptide stability and biological activity. In hyperglycemic conditions, VEGF expression is typically increased, leading to vascular permeability and inflammation^60^. In the zebrafish DR model, Peptide 1 significantly suppressed glucose-induced VEGF overexpression, while restoring Tie2 and Angiopoietin-1 expression, both of which were downregulated under hyperglycemic stress. These findings suggest that Peptide 1 may promote vascular stabilization by concurrently inhibiting VEGF signaling and enhancing Tie2/Ang-1 activity. Restoration of Angiopoietin-1/Tie2 signaling has been reported to maintain blood–retinal barrier integrity, reduce vascular leakage, and mitigate inflammation, thereby alleviating DR progression^61^. These molecular effects underscore the potential of Peptide 1 as a therapeutic candidate for DR. This study also reaffirmed the zebrafish DR model as a rapid and cost-effective platform for drug screening compared to conventional mammalian systems^62,63^. Given that the zebrafish retina is morphologically similar to the human retina, it has been reaffirmed as an ideal model for ophthalmic disease research. These findings emphasize the value of the zebrafish model not only for toxicity screening but also for therapeutic discovery^49,64^. Moreover, zebrafish offer advantages such as rapid cultivation and a cost-efficient experimental environment, enabling faster validation of drug candidates^65^. In conclusion, Peptide 1 emerged as the most promising candidate, combining low toxicity with vascular protective efficacy. Nonetheless, this study has limitations. The experiments were conducted in zebrafish only, and efficacy was not evaluated in mammalian models. Moreover, detailed structural analyses of the peptides were not performed, and the molecular mechanisms underlying their effects require further clarification. Future studies addressing these limitations, including sequence optimization and validation in higher-order models, will be essential to determine the translational potential of peptide-based therapeutics for DR.

## Materials and methods

### Zebrafish mating

Adult zebrafish were obtained from the Korea Zebrafish Disease Modeling Center (ZCDM) and maintained in the ZebTEC housing system (Tecniplast, Varese, Italy). As embryos beyond three days post-fertilization (dpf) are classified as vertebrates, all zebrafish protocols received approval from the Institutional Animal Care and Use Committee (IACUC) of Inje University Busan Paik Hospital (IJUBPH_2020-010-01). All procedures were conducted in accordance with relevant guidelines and regulations. The developmental stages of embryos were recorded in hours post-fertilization (hpf). This study is reported in accordance with the ARRIVE guidelines.

### Peptide design and preparation

Three test peptides (Peptides 1–3) were synthesized based on sequence modifications of a type II collagen-derived peptide (Patent Application No. 10-2020-0060397). The peptides were produced at > 95% purity and exhibited favorable physicochemical properties, including high thermal stability at 40 °C for 7 days (> 97% remaining) and improved aqueous solubility (5.8–500 mg/ml). For zebrafish experiments, peptides were freshly dissolved in Instant Ocean before administration. Since the toxicity profiles of these novel peptides in zebrafish had not been established, the test concentrations (50, 100, 200, and 400 µg/ml) were selected with reference to aflibercept concentrations previously evaluated in zebrafish studies^49^. This exploratory range was intended to encompass both non-toxic and potentially pharmacologically relevant levels.

### Morphological changes observation

Embryos obtained from mating adult zebrafish were selected 3–4 h after fertilization, ensuring that only normally fertilized embryos with regular morphology were used for the experiment. Embryos showing signs of developmental delay, unfertilized eggs, or morphological abnormalities were excluded from the study. The embryos were placed in 24-well plates with 5 embryos per well, and Peptide 1, Peptide 2, and Peptide 3 were diluted in Instant Ocean at concentrations of 50, 100, 200, and 400 µg/ml. A sample size of five embryos per group was selected based on prior zebrafish studies demonstrating sufficient statistical power for detecting morphological and vascular changes under similar experimental conditions. The survival rate, differentiation rate, and morphological changes of the zebrafish embryos were observed. To prevent contamination from the water, the treatment solution was replaced every 24 h. To monitor early developmental stages, photographs were taken using an optical microscope at 24, 48, 72, and 96 h post-fertilization. The number of embryos showing differentiation or death was counted and analyzed as a percentage compared to the control group. Embryos were randomly assigned to treatment groups to minimize allocation bias.

### Teratological changes observation

Teratological changes in zebrafish were observed based on predefined parameters. Photographs of deformities such as collapse of the fertilized egg (CPFE), yolk sac edema (YSE), pericardial edema (PE), bent tail (BT), and head edema (HE) were captured using a Motic (AE31E) microscope. The number of occurrences for each deformity was counted and analyzed as a percentage compared to the control group.

### Cardiotoxicity test

At 2 days post-fertilization (dpf), the chorion was removed from zebrafish embryos, and the embryos were transferred to 24-well plates (5 embryos per well in 1 mL of solution). Peptides 1, 2, and 3 were diluted in Instant Ocean to final concentrations of 20, 100, 200, and 400 µg/mL and applied for 1.5 h. After treatment, heart rates were measured by counting the number of beats over 10 s under a Motic AE31E microscope. Data are presented as mean beats per 10 s.

### Induction of zebrafish diabetic retinopathy model and drug treatment, hyaloid-retina vessel diameter measurement

On day 3 post-fertilization, zebrafish embryos with normal differentiation were treated with 130 mM glucose in a 24-well plate (5 embryos per well) to induce a diabetic retinopathy model. Peptides 1, 2, and 3 were administered at concentrations of 20, 100, 200, and 400 µg/ml. On day 6 post-fertilization, embryos were fixed with 4% paraformaldehyde and the hyaloid-retina vessels were isolated. Further experimental details can be found in previous studies.

### Genetic changes observation

The total RNA were extracted from the eyes at 6 dpf using the mRNA kit (PureLink™ RNA Mini Kit, Ambion). The mRNA was measured with a NanoDrop spectrophotometer (Thermo Scientific, Madison, WI, USA) for quantitative analysis and a cDNA synthesis with a SuperScript VILO cDNA Synthesis Kit (Thermo Scientific, Carlsbad, CA, USA). The cDNA was amplified by PCR with zebrafish primer GAPDH, VEGF, Tie2 and Ang-1 with SYBR Green Master Mix (Thermo Scientific, Carlsbad, CA, USA). The mRNA was detected by ViiA7 Real-Time PCR system (Thermo Scientific, Carlsbad, CA, USA). The values were normalized to GAPDH expression. Samples were analyzed in triplicate. The sequences of the primers used in this study are listed in Table 3.

### Animal euthanasia

At the end of all experimental procedures, zebrafish embryos were euthanized by immersion in an overdose solution of tricaine methanesulfonate (MS-222, 4 g/L; Sigma-Aldrich, St. Louis, MO, USA) prepared in system water. To prevent cross-contamination, embryos were immersed in the anesthetic solution in individual containers. Exposure continued until opercular movement had completely ceased, after which embryos remained in the anesthetic solution for at least 10 additional minutes to ensure death. Death was confirmed by the absence of a heartbeat under a microscope and no response to gentle tactile stimulation with fine forceps. All euthanasia procedures were conducted under the approval of the Institutional Animal Care and Use Committee (IACUC) of Inje University Busan Paik Hospital (IJUBPH_2020-010-01) and in accordance with relevant institutional guidelines and the AVMA Guidelines for the Euthanasia of Animals.

### Statistical analysis

The data were analyzed using one-way ANOVA with Tukey’s post hoc analysis by PRISM software (GraphPad, San Diego, CA, USA). A *p-*value < 0.05 was considered statistically significant.

## Data Availability

The datasets used and/or analysed during the current study available from the corresponding author on reasonable request.
